# Frequency Domain Analysis and Precision Realization in Deterministic Figuring of Ultra-Precision Shaft Parts

**DOI:** 10.3390/ma13204561

**Published:** 2020-10-14

**Authors:** Zizhou Sun, Hao Hu, Yifan Dai, Chaoliang Guan, Guipeng Tie, Yang Ou

**Affiliations:** 1 College of Intelligent Science and Technology, National University of Defense Technology, Changsha 410073, China; 18302996932@163.com (Z.S.); huhao07@nudt.edu.cn (H.H.); chlguan@nudt.edu.cn (C.G.); tieguipeng@163.com (G.T.); yang_ou17@163.com (Y.O.); 2 Hunan Key Laboratory of Ultra-Precision Machining Technology, Changsha 410073, China; 3 Laboratory of Science and Technology on Integrated Logistics Support, National University of Defense Technology, Changsha 410073, China

**Keywords:** deterministic figuring, aerostatic spindle, filtering, CFD analysis, PSD curve

## Abstract

An aerostatic spindle is a core component in ultra-precision machine tools. The rotor of the spindle has extremely high manufacturing accuracy, which cannot be directly achieved via traditional machining, but always via manual grinding. The deterministic figuring theory is introduced into the machining of shaft parts, which overcomes many shortcomings of manual grinding. The manufacturing error of the shaft’s surface contains different frequency components, which have different effects on its working performance and the figuring process. Because the deterministic figuring method can only correct the error within a limited frequency range, in order to ensure high efficiency and high precision of the figuring process, we need to use reasonable filtering parameters to filter out the error with unnecessary frequencies. In this paper, the influence of contour error with different frequencies and amplitudes on the air film are analyzed using computational fluid dynamics (CFD) software, and the amplitude–frequency analysis as a function of the power spectral density (PSD) characteristic curve is used to study the filtering parameters of the measured data. After the figuring experiment using the filtering parameters obtained from the analysis, the average roundness of the shaft converged from 0.419 μm to 0.101 μm, and the cylindricity converged from 0.76 μm to 0.35 μm. The precision reached the level of manual grinding, which proves the rationality of the analysis using filtering parameters in a shaft’s deterministic figuring.

## 1. Introduction

Ultra-precision aerostatic spindles are widely used in various ultra-precision machining and measuring equipment due to their advantages of high rotating accuracy, low friction, low noise, etc. [1]. With the development of ultra-precision machining technology, the required precision of some ultra-precision parts has stepped up to the level of sub-micron or even sub-nanometer, which puts forward higher requirements for the machining accuracy of ultra-precision machine tools. The spindle copies its own rotating error onto the workpiece during machining; thus, the spindle’s rotating accuracy is an important evaluation index of the machine tool’s performance [2]. The radial rotating accuracy of the spindles of Nanoform^TM^ series ultra-precision machine tools produced by the Precitech^®^ and Nanotech^TM^ series from Moore^®^ has reached 25 nm, and the surface form error of the workpiece can reach 0.1 μm. The rotating accuracy of the aerostatic spindle is affected by the machining accuracy of the bearing and the spindle rotor. The RONt (roundness error) and the CYLt (cylindricity error) at the rotor’s journal are the main factors affecting the rotating accuracy [3]. According to the actual production, if the rotating accuracy of the spindle reaches 25 nm, the RONt of the journal should reach 0.1 μm and the CYLt should be within 1 μm. Many scholars have studied the fundamental problems in traditional machining. For example, Wojciechowski constructed a new model that takes into account the chip thickness accumulation phenomenon, which can better predict the cutting force in the process of precise micro-cutting [4]; Rudrapati and Aleksandrova studied parameter optimization in cylindrical grinding [5,6]. Due to the limit precision of traditional cylindrical machining, an ultra-precision cylindrical grinder can only reach a RONt of 0.2–0.3 μm. A further improvement in precision can only be achieved via manual grinding and polishing, which has low efficiency and high production cost, which cannot meet the requirements of mass production. At the same time, manual polishing highly depends on the experience of technicians, and the uncertainty of the process is inevitable. It is very difficult to further improve the manufacturing accuracy of a spindle rotor.

By introducing the deterministic figuring theory for plane and freeform surfaces into the machining of shaft workpieces, the automated machining of the shaft’s surface with high precision and high efficiency can be realized theoretically. The deterministic figuring is based on the CCOS (computer-controlled optical surfacing) principle [7,8], under the condition of ensuring an accurate removal function and initial surface form error, whereas the dwell time of the removal function at each position on the shaft’s surface is calculated using an accurate algorithm, which is accurately controlled by the CNC (Computer numerical Control) system. When the shaft workpiece is servo-controlled by the CNC system and rotates at nonuniform speed, the high-error area on the workpiece dwells in the polishing area for a longer time while the low error area dwells for a shorter time. The geometric error of the shaft can be corrected deterministically. Our previous work mainly studied the figuring ability and generation mechanism of the removal function in deterministic figuring on shaft parts [9]. By modifying the process parameters, the original removal function is optimized, which improves the convergence accuracy and convergence efficiency, and reduces the influence of positioning error on the accuracy’s convergence. 

In deterministic figuring, one removal function has limited figuring ability, which cannot correct the errors of all spatial frequency components. However, the frequency components of the manufacturing error on the shaft’s surface are very rich, and the redundant frequency components affect the dwell time’s calculation. At the same time, the error components of different frequencies have different effects on the working performance of the shaft. In order to filter out errors with redundant frequencies, we need to filter the shaft’s surface, but there is no basis for the selection of filtering parameters for an aerostatic spindle’s rotor. Kim and Wang [10,11] studied the influence of the rotor’s form error with low-frequency components on the rotating accuracy; Wang [12] analyzed the influence of the bearing’s waviness error on the static characteristics of aerostatic spindle on the basis of an assumption that the spindle rotor has no form error.

Therefore, this paper uses CFD (computational fluid dynamics) simulations to analyze the film’s flow field of the aerostatic spindle, combining the amplitude–frequency characteristics of the actual circular contour to study the influence of the circular form error with different frequencies, before setting reasonable filtering parameters for the experimental shaft’s surface data, which are used to guide the deterministic figuring and achieve a RONt of 0.1 μm.

## 2. Brief Introduction of Deterministic Figuring for Shaft’s Cylindrical Surface

Precision finishing for steel shaft parts with a flexible processing method offers manufacturers an alternative machining method to traditional turning and grinding [13]. To improve finishing efficiency and surface quality, the vibration assistance method has been used in some abrasive finishing processes, such as vibration-assisted magnetic abrasive finishing [14,15], vibration-assisted belt abrasive finishing [16], and ultrasonic finishing [17].

**Figure 1:**
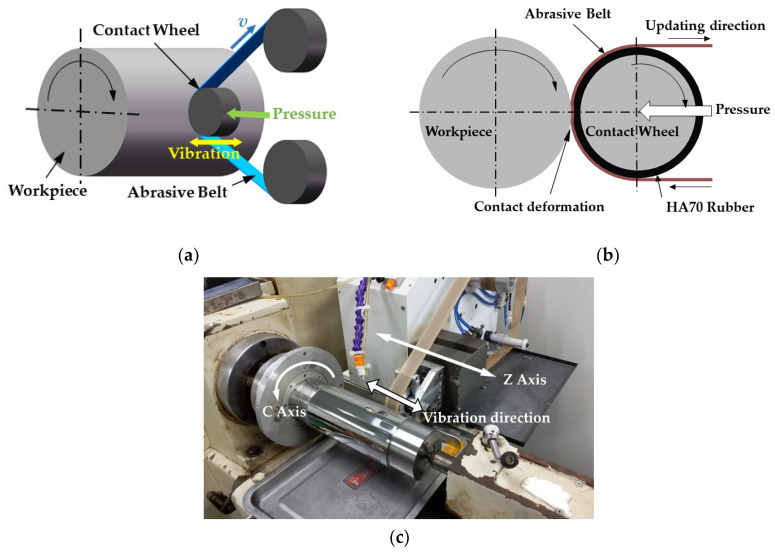
Schematic diagram of deterministic grinding with vibration-assisted abrasive belt: (**a**) three-dimensional schematic diagram; (**b**) two-dimensional schematic diagram; (**c**) actual diagram of the system.

The principle of a shaft’s deterministic figuring using a vibration-assisted abrasive belt is shown in Figure 1. The axis of the contact wheel is parallel to the axis of the workpiece, whereas the outer ring’s material of the contact wheel is made of rubber with a hardness of HA70 and is covered with the abrasive belt. The contact wheel is fixed on a cylinder which can push the contact wheel to contact the workpiece with a constant pressure, resulting in deformation and forming one rectangular contact area. Vibration in the axial direction occurs at a certain frequency to provide the main movement of material removal. At the same time, depending on the linkage of the *C*-axis and *Z*-axis, the contact wheel can pass through the whole shaft’s surface. When the machining parameters such as the pressure of the cylinder, the vibrating frequency of the contact wheel, and the updating speed of the abrasive belt remain unchanged, because the contact wheel is always in contact with the workpiece due to pressure during the figuring process, a longer dwell time of the shaft’s surface in the contact area leads to more material being removed, and there is a linear relationship between the amount of removed material and the length of dwell time. Therefore, as long as the servo *C*-axis and *Z*-axis are controlled to move at a nonuniform speed, the high-error positions of the workpiece surface can remain in the contact area for longer time, thereby causing more material removal, while the low-error positions remain for a shorter time, with less material being removed. In this way, the contour of shaft parts is gradually corrected deterministically to approach an ideal cylindrical surface. Furthermore, due to the polishing effect of the abrasive belt, the surface quality of the workpiece can be improved during the improvement of shape accuracy in the process of figuring.

In order to control the grinding state of the abrasive belt and ensure the stable removal efficiency of the material during the figuring process, the belt updates at a certain speed to maintain the grinding ability of abrasive grits in the contact area at a stable level. The removal function is a distribution matrix of the removal amount obtained by measuring the removal area generated by the contact wheel on the shaft’s surface. As shown in Equation (1), during the process of deterministic figuring, the two-dimensional convolution of the removal function matrix *R(x, y)* and the dwell time matrix *T(x, y)* is the matrix of total removal amount *E(x, y)*.

(1) E(x, y) = R(x, y)*T(x, y).

**Figure 2:**
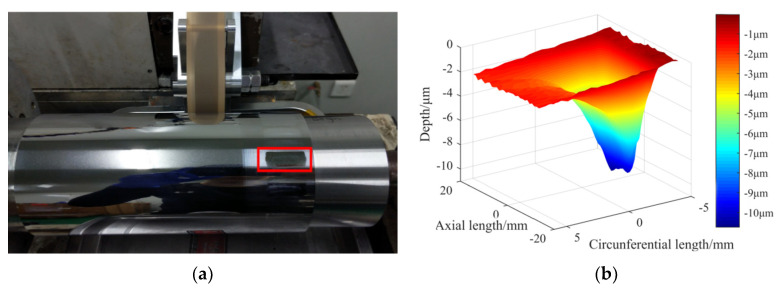
The removal function of deterministic grinding with a vibration-assisted abrasive belt: (**a**) actual appearance; (**b**) the extracted three-dimensional morphology.

A removal function according to the above processing method has a three-dimensional shape, as shown in Figure 2, which can be decomposed into two directions of the contour along the circumferential direction and along the axial direction.

## 3. Filtering Processing of Measurement Data of the Cylindrical Surface

**Figure 3:**
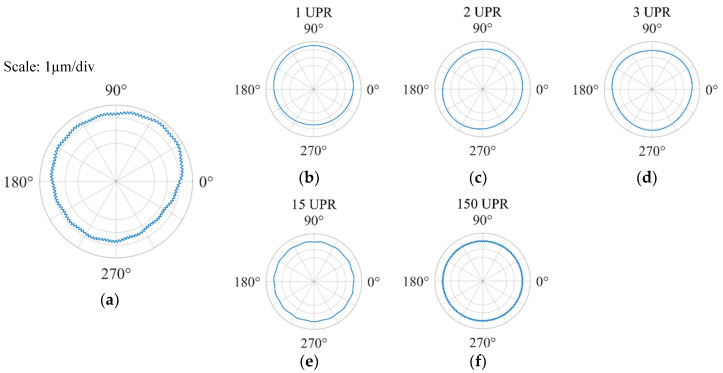
A schematic diagram of the contours with the harmonics containing different frequency components: (**a**) overall circular contour; (**b**) harmonic error with 1 UPR; (**c**) harmonic error with 2 UPR; (**d**) harmonic error with 3 UPR; (**e**) harmonic error with 15 UPR; (**f**) harmonic error with 150 UPR.

The manufacturing errors of the workpiece after machining include a geometry error, waviness, and surface roughness, which correspond to the low-frequency band, medium-frequency band, and high-frequency band, respectively. Through the Fourier series, we know that any continuous measured signal can be represented by an infinite series composed of a sine function and cosine function. A nominally round part can be viewed as comprising different harmonics which are specified in terms of undulations per revolution (UPR) [18], as explained in Figure 3.

The cutoff frequency and cutoff wavelength of harmonics have the following relationship:

ω_c_ = πD/λ, (2)

where *ω_c_* represents the cutoff frequency, *λ* represents the cutoff wavelength of the contour, and *D* represents the diameter of the circle.

The RONt and CYLt of the shaft can be measured using a CMM (coordinate-measuring machine), cylindricity meter, and other equipment. The measuring equipment we used was a Talyrond^TM^ 565H cylindricity meter made by Taylor Hobson^®^. The cylindricity meter adopts the structure of the spindle rotating while the measuring arm is not rotating. The measuring surface is contacted by the ruby probe, and the contour’s offset signal is output by a precise sensor. The measurement uncertainty is less than 30 nm, and the maximum number of sampling points of one circle is 18,000 points. Through processing using software, closed circular contour data can be obtained.

The traditional measurement and evaluation indices of shaft parts mainly include RONt, CYLt, amd runout. The cylindricity meter we used can display the value and angular position of RONp (peak value of the roundness error) and RONv (valley value of roundness error) on one circular section. However, in deterministic figuring, only by obtaining a certain surface error distribution and surface error frequency components can we know where to remove more and where to remove less, before analyzing the quality of the figuring with respect to the removal function we used. Therefore, the evaluation method used for the traditional cylindrical surface error obviously cannot meet the requirements. In the process of deterministic figuring, we pay more attention to the macro shape contour rather than the roughness for the following reasons:

1. Because the amplitude of the macro form error is larger, it is the main component of the RONt, which has the greatest impact on the rotating accuracy, while the amplitude of the high-frequency error is always small;
2. In deterministic figuring, one removal function has limited figuring ability, and it has no correction effect on the error’s frequencies which exceed the upper limit of its figuring ability. Higher-frequency errors can only be removed by removal functions with stronger figuring ability, which puts forward high requirements for the positioning accuracy and dynamic characteristics of machine tools, whereby the actual feasibility is low, as later discussed in detail.

Therefore, we do not consider the correction of high-frequency error components in the current state. If the shaft has a rough surface texture, the macro geometry of the surface may be concealed by its surface roughness, which affects our accurate judgment of the macro shape. If the measured data is filtered reasonably, it is helpful to separate the useful data and the redundant data on the circular contour. The most widely used filter for surface profile analysis is the Gaussian filter for its good amplitude transmission characteristics [18]. By filtering the raw contour data with a Gaussian low-pass filter at a certain cutoff frequency, high-frequency errors can be filtered out and the macro contour data we need can be obtained, which reduces the difficulty of convergence on the premise of ensuring precision. Therefore, taking an AISI 1045 steel shaft with a diameter of 100 mm after cylindrical grinding as an example, the filtering basis of the measured cylindrical surface data is given in this section through the analysis of a CFD simulation describing the film’s flow field and the amplitude–frequency characteristics of the cylindrical surface.

### 3.1. Selection of Filter Parameters According to CFD Simulation of Film’s Flow Field 

**Figure 4:**
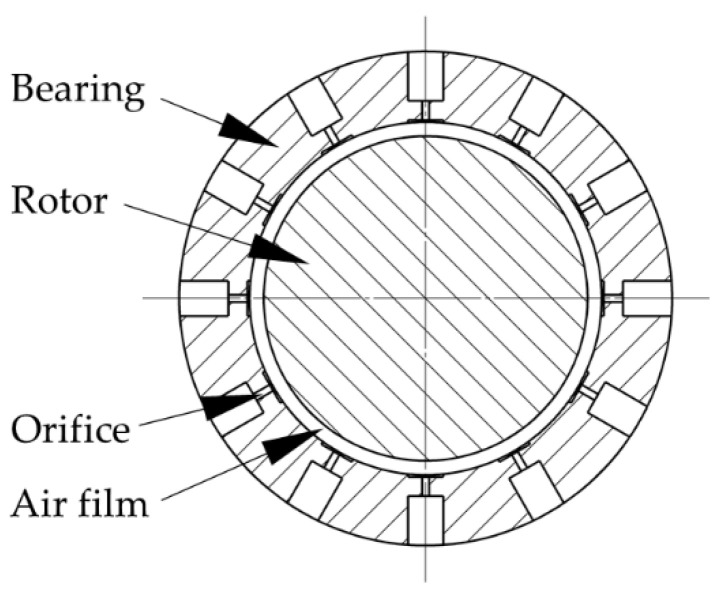
Schematic diagram of the radial section of the orifice throttling aerostatic spindle.

The reason why the aerostatic spindle has higher rotating accuracy than its manufacturing error is that the air film of the aerostatic spindle has an effect called error homogenization. At present, the main throttling forms of aerostatic spindles include orifice throttling, slit throttling, and porous throttling. As a typical throttling method of aerostatic spindles, orifice throttling has been widely used. The radial section diagram of an aerostatic spindle with orifice throttling holes is shown in Figure 4. Throttle holes are distributed evenly on the bearing of the spindle along the circumferential direction. Compressed air enters the gap between the bearing and the rotor through the orifice to form a layer of air film, of which the thickness is usually about 15–25 μm. 

Due to the form error on the rotor’s surface, when the rotor rotates, the thickness of the air film changes, and the corresponding supporting force of the air film changes, which leads to a change in the resultant forces of the film acting on the rotor, causing it to have radial displacement, and finally forming the radial rotating error.

**Figure 5:**
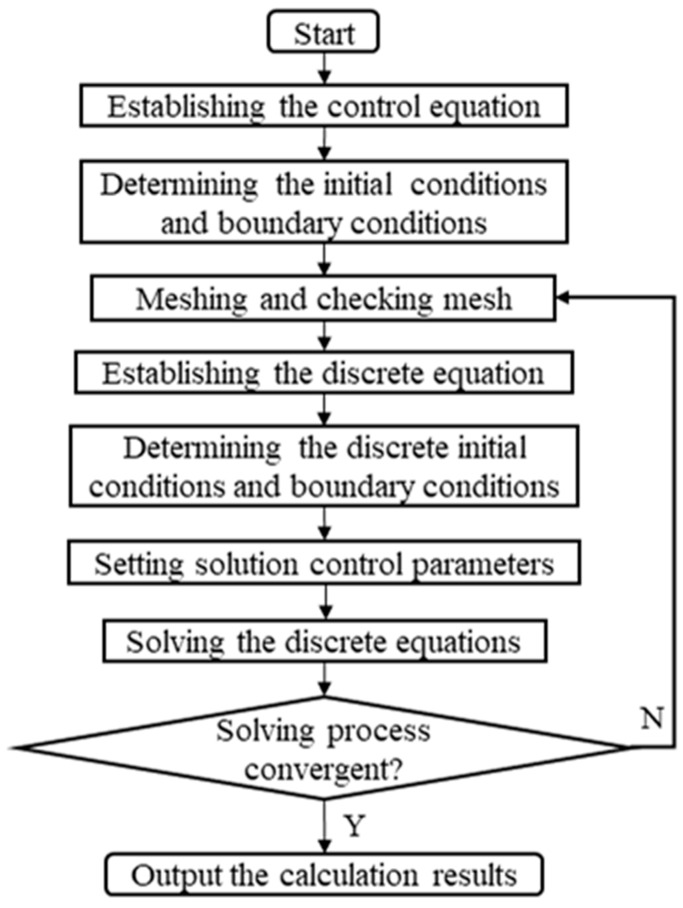
Flow chart of computational fluid dynamics (CFD) calculation.

Theoretically speaking, the homogenization effect improves with an increase in the number of circumferential orifices; however, too many orifices reduce the radial stiffness of the spindle, while too few orifices make the film not uneven enough, which affects the effect of error homogenization. Generally, 8–12 orifices are selected on one circle and arranged in multiple rows to improve its bearing capacity and stability [19]. CFD combines modern fluid mechanics, numerical mathematics, and computer science, and it is widely used in the simulation of various fluids. In this subsection, the CFD module of ANSYS (ANSYS Inc., Pittsburgh, PA, USA) is used to analyze the film’s flow field influenced by the contour error of the rotor. The process of CFD simulation is shown in Figure 5.

**Figure 6:**
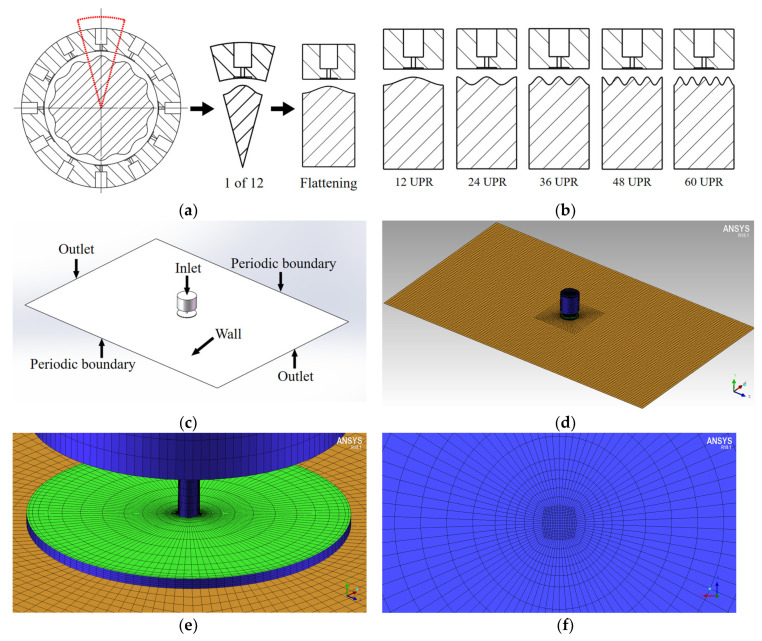
Preprocessing of CFD analysis: (**a**) schematic diagram of the simplified structure; (**b**) schematic diagram of outflow region with different error frequencies; (**c**) model of inner flow region and setting of boundary conditions; (**d**) global structured mesh by ICEM^TM^; (**e**) inflation at the edge; (**f**) inflation at the orifice.

In order to calculate efficiently and accurately, we selected 1/12 of the spindle in the circumferential direction, flattening the arc contour into a plane contour, then used SolidWorks (Dassault Systèmes SolidWorks Corporation, Concord, MA, USA) to draw the air film fluid domain model with the rotor’s contour error. After the model was built, the Fluent module was used for CFD analysis and the CFD post module was used for postprocessing. In this example, the orifice’s diameter was 0.3 mm, the orifice chamber’s diameter was 3 mm, the thickness of the film was 15 μm, the inlet pressure was set as 0.6 MPa, and the outlet pressure was set as 1 bar (101,325 Pa). Moreover, the boundary conditions of the flow field were set as shown in Figure 6.

In order to more clearly show the contrast effect of qualitative analysis, and in order to make the cosine curve on the 1/12 model continuous and derivable on the boundary, we set the error in the same amplitude (2 μm), and the error frequency was preferentially set to 12 UPR, 24 UPR, 36 UPR, 48 UPR, and 60 UPR for numerical calculation. The mesh was divided into a structured mesh using the ICEM^TM^ module in ANSYS. The preparatory work of a structured mesh is time-consuming, but the speed of mesh generation is fast, and the generated mesh allows easier convergence in subsequent calculations. At the same time, the quality of a structured mesh is better than that of an unstructured mesh under the same number of elements. In the Fluent module, we chose to use the K–ε turbulence model. The calculation method used the SIMPLE algorithm, and the under-relaxation factors of pressure, density, body forces, and momentum were set to 0.2, 0.8, 0.8, and 0.6, respectively [20]. A slower convergence speed was used in exchange for a higher convergence accuracy.

**Figure 7:**
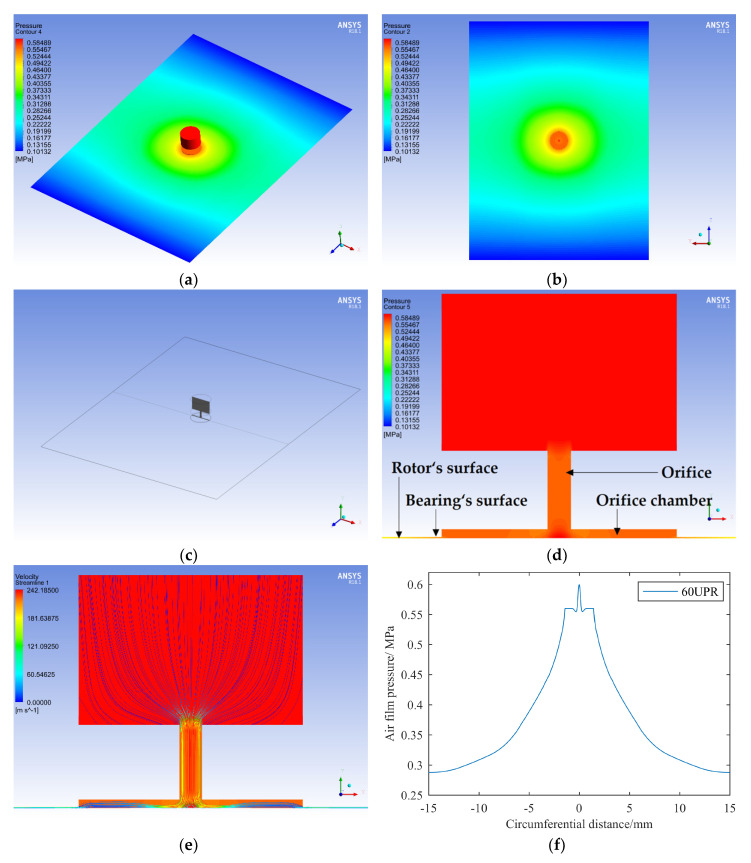
Analysis of the film’s flow field with an error frequency of 60 undulations per revolution (UPR): (**a**) film pressure distribution of the whole flow field; (**b**) film pressure distribution on rotor surface; (**c**) schematic diagram of radial section at orifice; (**d**) pressure distribution on radial section; (**e**) streamline distribution on radial section; (**f**) pressure distribution curve on the rotor surface along the circumferential direction.

We inserted a radial section through the center of the orifice, as shown in Figure 7c, and then focused on analyzing the pressure distribution on the intersection line between the above section and the shaft’s surface. According to the results, the film’s pressure was the maximum at the position right below the orifice, which was close to the inlet pressure. The air pressure in the rest position of the orifice chamber was basically constant, and the pressure gradually decreased toward the outlet surface and periodic boundary.

Next, the film’s flow field with errors under different UPR was analyzed. When UPR = 0 (when there was no roundness error on the circular contour), the average pressure at the orifice chamber was the minimum. We defined the relative variation ratio of the film pressure *η* as follows:

(3) $$\eta =\frac{p_{n}-p_{0}}{p_{0}},$$

where *p_n_* is the average pressure at the orifice chamber when the contour error’s frequency is *n*UPR, and *p_0_* is the average pressure at the orifice chamber without error.

**Figure 8:**
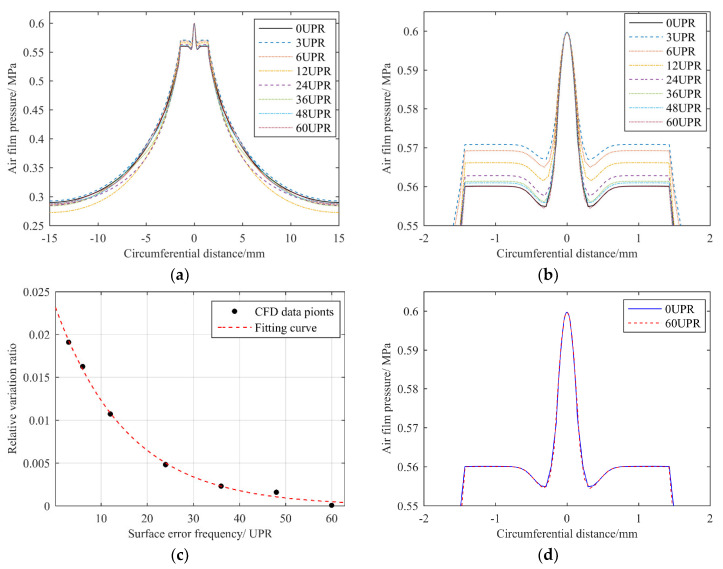
Comparison of CFD results of contour error in different frequencies: (**a**) pressure distribution curve on the rotor’s surface along the circumferential direction; (**b**) local amplified pressure distribution at the position of orifice chamber; (**c**) relative variation ratio of the film pressure under different contour error frequencies; (**d**) comparison of pressure distribution at the orifice chamber with a contour error frequency of 0 UPR and 60 UPR.

When a contour error occurred, the high point of the error made the film thickness narrow while the corresponding pressure increased. However, with an increase in the error frequency, the passivation effect of the air film on the contour’s error became more obvious, and η gradually decreased, as shown in Figure 8c, where the experimental data points could be well fitted by an exponential curve. When the error frequency reached 48 UPR, η was less than 0.1%; when the frequency reached 60 UPR, η was close to 0, and it could even be ignored as a calculation error.

Therefore, we can draw a conclusion that a higher error frequency has less influence on the film’s flow field when the error amplitude is the same. The influence of a contour error with a frequency of 60 UPR on the pressure distribution at the orifice chamber can be ignored. We defined the cutoff line as 1% of the pressure’s relative variation ratio at the orifice chamber, and the corresponding cutoff frequency was about 13.5 UPR.

**Figure 9:**
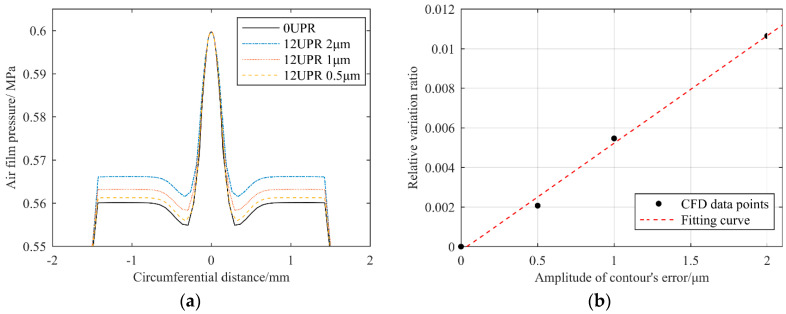
Comparison of CFD results for different error amplitudes when the frequency was fixed at 12 UPR: (**a**) comparison of pressure distribution at the orifice chamber; (**b**) relative variation ratio of the film’s pressure.

In fact, in order to more clearly reflect the influence of error frequencies on the flow field’s pressure, we set the amplitude of errors to 2 μm at all frequencies. However, in fact, the error’s amplitude cannot be that large, as the amplitude of the error decreases with an increase in frequency, as shown in the analysis in the next section. For example, when the error frequency was fixed at 12 UPR, we set different error amplitudes for CFD calculation, and the results are shown in Figure 9.

It can be seen that, with a decrease in error amplitude, the influence on the pressure at the orifice chamber gradually decreased at the same frequency, and the data points could be approximately linearly fitted. Therefore, the cutoff frequency of 13.5 UPR is relatively conservative.

### 3.2. Analysis of Filtering Parameters According to Amplitude–Frequency Characteristics

#### 3.2.1. Figuring Ability of the Removal Function in Deterministic Machining

The removal function of deterministic figuring can only correct the error components within a certain frequency, and the error with a frequency band beyond the range of its figuring ability is meaningless to the removal function itself. The figuring ability of one removal function depends on its contour characteristics, which can be described by the normalized amplitude spectrum of the Fourier transform of the contour. The corresponding frequency at 5–10% of the maximum amplitude is defined as the cutoff frequency, and the error components lower than the cutoff frequency can be theoretically corrected using the removal function [21]. Generally, the removal function with a low cutoff frequency has a smoother contour, which is suitable for correcting error components with longer spatial wavelengths, but incapable of correcting high-frequency error components. The removal function with a high cutoff frequency has a sharper contour, which can be used to correct both low- and high-frequency components. However, it has very high requirements in terms of positioning accuracy of the machine tool; once the removal function is not aligned at the machining starting point, the specified amount may be removed at the wrong position, resulting in a small amount of removal at high-error points and a large amount at low-error points, resulting in a failure of the accuracy to converge. At the same time, if we blindly pursue removal functions with high figuring ability, a large local acceleration is generated during the calculation of dwell time, which puts forward high requirements in terms of the dynamic performance of the machine tool, and the actual machining results cannot meet theoretical expectations.

#### 3.2.2. Contour Amplitude–Frequency Analysis According to Power Spectral Density (PSD) Characteristic Curve

In the process of developing the NIF (National Ignition Facility Project), the LLNL (Lawrence Livermore National Laboratory) proposed a method to evaluate the optical surface error with the PSD (power spectral density) characteristic curve [22,23], which became a new method to evaluate the surface error of optical parts in the optical international standard ISO 10110 (promulgated in 1997). The essence of PSD is Fourier spectrum analysis. Through Fourier transform, the spatial frequency distribution of the wavefront error of the optical element can be given quantitatively, so as to determine the influence of each frequency component.

**Figure 10:**
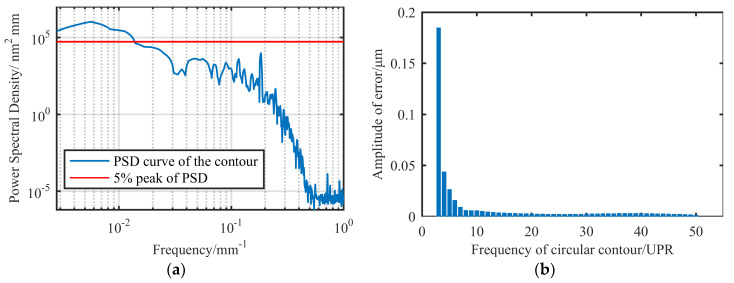
Analysis of amplitude–frequency characteristics of contour: (**a**) power spectral density (PSD) characteristic curve; (**b**) error amplitude of different frequency components.

We exported the data measured by the cylindricity meter, expanded it into a rectangular surface along a generatrix on the cylindrical surface, and then performed PSD analysis on the contour along the circumferential direction. The results are shown in Figure 10.

It can be seen in Figure 10a that the amplitude of contour error decreased with an increase in frequency. In general, the frequency corresponding to 5–10% of the maximum power density is called the cutoff frequency [24], and the components exceeding the cutoff frequency do not need to be considered in the single figuring process. Taking 5% of the peak value as the cutoff line, we obtained the cutoff frequency in the circumferential direction of the surface as 0.1412 mm^−1^, and the corresponding cutoff wavelength was 70.82 mm. According to Equation (2), the cutoff harmonic frequency was 5.1 UPR.

In fact, by further analyzing the measurement contour, it can be obtained, as shown in Figure 10b, that the error amplitude of 7 UPR was already less than 0.01 μm, which was less than 5% of the corresponding amplitude of 3 UPR. Therefore, from the perspective of the PSD curve, the main contribution to the RONt is the component between 3 and 6 UPR. Figure 10b shows a trend of rapid decay before exceeding 10 UPR, where it enters a stage of slow decay with amplitudes of less than 0.005 μm.

## 4. Experimental Details

### 4.1. Simulated Machining Process Using Different Filtering Parameters

**Figure 11:**
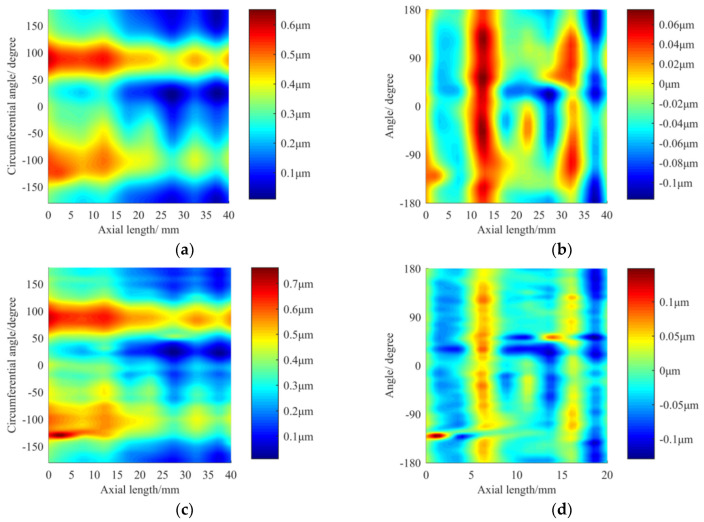
Surface morphology before and after simulated machining under different filtering parameters: (**a**) 5 UPR filtering before machining; (**b**) 5 UPR filtering after machining; (**c**) 15 UPR filtering before machining; (**d**) 15 UPR filtering after machining.

According to the results of the analysis in the previous section, we obtained a filtering basis for the shaft’s measured data. Next, we performed simulated machining on the measured cylindrical surface. We performed 1–5 UPR filtering and 1–15 UPR filtering on the raw measurement data; then, we simulated the machining process using the same removal function and compared the processing results of different filtering parameters. The process and results of simulated machining are shown in Figure 11 and Table 1.

The simulated machining process needed the measured surface data *S (x, y)* of the shaft part to be machined and the removal function *R (x, y)*, which was extracted from the sample workpiece (the sample workpiece had the same material and same diameter as the shaft to be machined). The dwell time matrix *T (x, y)* can be solved using the pulse iteration method [9]. Because the deterministic figuring process requires that every position on the machined surface passes through the grinding area (i.e., the removal function reaches each position of the measuring surface), the two-dimensional convolution of the removal function *R (x, y)* and dwell time *T (x, y)* can represent the theoretical removal amount of each position on the shaft’s surface. Therefore, the convergent effect of the accuracy can be theoretically judged using the simulation. The residual error matrix of the surface after machining *Re (x, y)* can be obtained using the following equation:

*Re (x, y)* = *S (x, y)* − *R (x, y)* × *T (x, y)*. (4)

It can be seen that, after using 1–5 UPR and 1–15 UPR Gaussian filtering, the high-frequency error component was effectively suppressed while the macro contour information was retained, and the average RONt after both simulation processes reached our goal. In order to remove the error in a wider frequency band within the figuring ability of the removal function, we finally decided to use 1–15 UPR Gaussian filtering on the surface to be processed, which not only ensured that the precision after processing met the requirements, but also enabled the error in a wider frequency band to be effectively corrected.

### 4.2. Details of Actual Experiment Process

**Figure 12:**
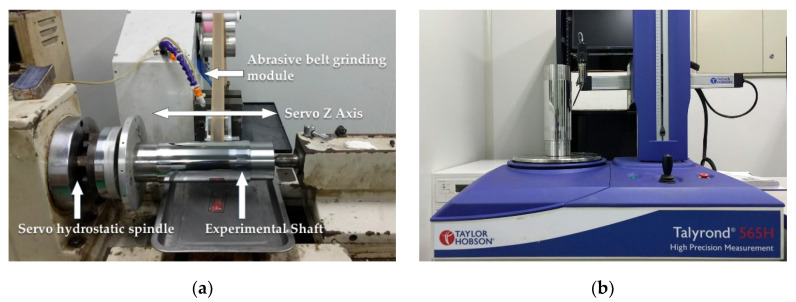
Actual figuring and measuring equipment of the experimental shaft: (**a**) modified lathe; (**b**) Talyrond^TM^ 565H cylindricity meter.

The actual equipment used for the experiment was a modified lathe, whose spindle was replaced by a hydrostatic spindle, while the servo control system was modified to meet the machining requirements. Details of the experimental shaft and machining parameters are shown in Figure 12, Table 2 and Table 3, respectively.

When measuring the surface data of the workpiece, a starting point needs to be marked. Before machining, the center of the contact wheel should be aligned with the previous starting point in order to ensure that the actual starting point of the machining is as close as possible to the starting point of the surface error matrix. Only in this way can the specified amount of material be removed at the specified position during the machining process.

During the grinding process, the material removed from the surface of the workpiece is taken away by the updating abrasive belt. Because abrasive wear and clogging of debris occur during grinding, the repeated use of abrasive belts seriously affects the stability of the removal function. Therefore, we selected an open-loop abrasive belt and updated it at a certain speed to ensure the stability of the removal function.

## 5. Results and Discussion

**Figure 13:**
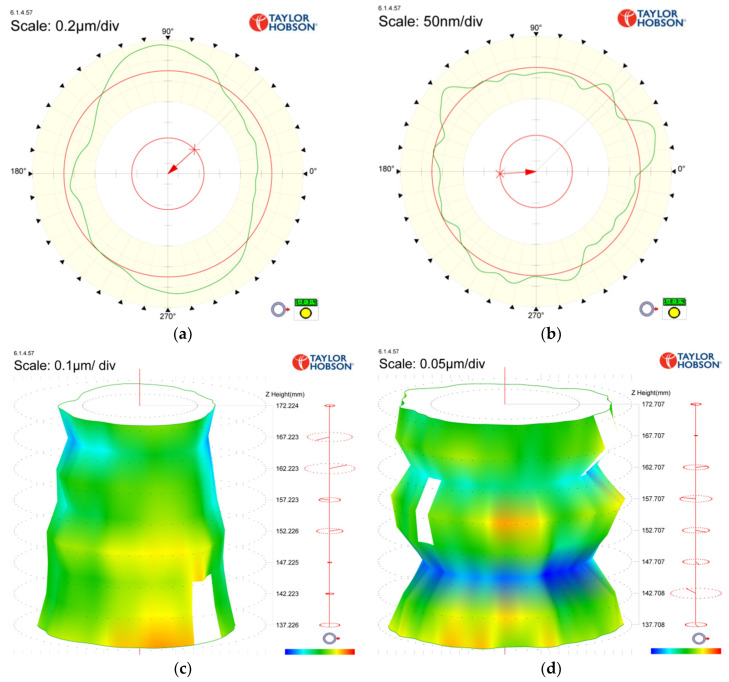
RONt and CYLt measurement results before and after machining: (**a**) RONt before machining in Section 6; (**b**) RONt after machining in Section 6; (**c**) CYLt before machining; (**d**) CYLt after machining.

Finally, 1–15 Gaussian UPR filtering was selected to perform the figuring experiment on the shaft with an effective length of 35 mm. The whole process took 25 min. The RONt and CYLt before and after processing are compared in Figure 13.

Since all circular contours change roughly the same before and after machining, Figure 13 does not show the RONt measurements of all circular contours before and after machining. Taking Figure 13a,b as examples, we can further explain the mechanism of deterministic figuring. As can be seen from Figure 13a, the positions near 90° and 270° were the high points of the contour error, while the positions near 0° and 180° were the low points of the contour error. During the process of figuring, when the servo spindle rotates with the workpiece to 90° and 270°, the servo spindle rotates more slowly, while the vibrating contact wheel grinds in the contact area for a longer time and removes more material. Moreover, when the workpiece is turned to the position near 0° and 180°, the servo spindle rotates with the workpiece as fast as possible to pass the contact area so that less material is removed. Therefore, the contour is gradually corrected from a shape close to an ellipse (see Figure 13a) to the shape shown in Figure 13b. According to Figure 13 and Table 4, after deterministic figuring on the cylindrical surface, the average RONt converged from 0.419 μm to 0.101 μm with a convergence ratio of approximately 4.15. The RONt of some sections exceeded 0.1 μm, achieving the precision of manual grinding. The CYLt converged from 0.76 μm to 0.35 μm with a convergence ratio of approximately 2.17. 

**Figure 14:**
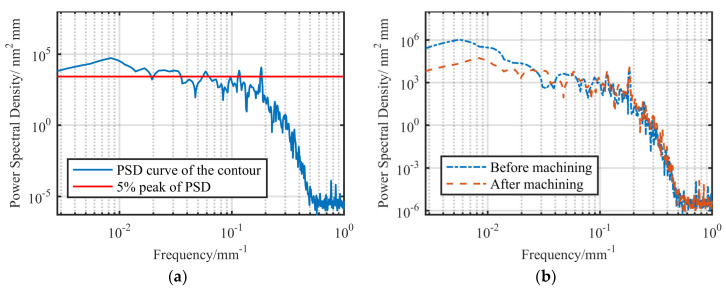
PSD analysis of peripheral contour after machining: (**a**) PSD characteristic curve after machining; (**b**) comparison of PSD characteristic curve before and after machining.

It can be seen from Figure 14 that the surface error after processing had obvious convergence in the low-frequency band, whereas the curves before and after machining show that the errors with frequency less than 0.04 mm^−1^ (<15.4 UPR) were effectively corrected, reaching the primary goal of correcting the macro contour error in the low-frequency band, while also verifying the necessity of filtering and the rationality of the filtering parameters. 

It is worth mentioning that the filtering method only filters the high-frequency error of the measured data, but does not filter the high-frequency error on the actual shaft’s surface. The correction of these high-frequency components is also skipped during actual machining. Therefore, contour errors with frequencies beyond the filtering range still exist before and after machining. For example, as shown in Figure 14b, there was an error peak at the frequency of about 0.2 mm^−1^ (61 UPR), the magnitude of which hardly changed before and after machining. However, according to the results of Section 3.1, the influence of the error at such frequency on the film’s flow field can be ignored; thus, it was not necessary to consider correcting the error at this frequency.

It can also be seen from Figure 14 that the cutoff frequency of the contour after machining reached 0.187 mm^−1^ (57.4 UPR), mainly because the amplitude of the low-frequency error was greatly corrected, and the relative amplitude of the high-frequency error increased. According to the definition of the cutoff frequency, it is higher after machining than before machining; Secondly, the amplitude of some high-frequency errors after processing exceeded that before processing; because the cutoff frequency of the removal function was higher than that of the original surface, the removal function reproduced its own frequencies on the cylindrical surface during figuring. This is one of the characteristics of deterministic figuring [25].

## 6. Conclusions

In this paper, a figuring experiment was performed on an experimental steel shaft by means of vibration-assisted abrasive belt deterministic grinding. According to the analysis of CFD on the air film’s flow field and amplitude–frequency characteristics of the measured data, the filtering parameters were given. Finally, simulations and practical experiments were carried out using the above parameters, and the expected results were obtained. The main conclusions are as follows:

- Error convergence could be achieved by deterministic figuring on the surface of the shaft. The average RONt converged from 0.419 μm to 0.101 μm with a convergence ratio of approximately 4.15, which surpasses the machining limit of ultra-precision cylindrical grinding. The roundness of some sections exceeded 0.1 μm, achieving the precision of manual grinding. The CYLt converged from 0.76 μm to 0.35 μm with a convergence ratio of approximately 2.17. The feasibility of the deterministic figuring method with a vibrating abrasive belt was verified by experiments, which shows its potential in the machining of ultra-precision shaft parts.
- By using CFD analysis in the film’s flow field and the analysis of amplitude–frequency characteristics according to measured data, the selection basis of filtering parameters was given. The results of simulations and experiments showed that it is necessary and reasonable to filter the measured data before machining.
- The influence of shaft contour error on the air film was analyzed using CFD software. The influence of shaft contour error on the air film pressure at the orifice decreased with an increase in error frequency. When the error frequency reached 48 UPR, the relative variation ratio of the air film was less than 0.1%, and the ratio was almost zero when the frequency reached 60 UPR. When the error frequency was fixed, the influence on the pressure at the orifice chamber gradually decreased with the decrease in error amplitude.
- After analyzing the amplitude–frequency characteristics of the measured data, it was found that the components mainly affecting the whole contour error are concentrated in the frequency band of less than 10 UPR. The error amplitude exceeding 7 UPR was less than 0.01 μm, i.e., less than 5% of the corresponding amplitude of 3 UPR, and the error amplitude with frequency exceeding 10 UPR was less than 0.005 μm.
- The deterministic figuring of ultra-precision shaft parts is a completely new field, and there are also many aspects to be studied for the deterministic figuring on the rotor of aerostatic spindles. For example, the influence of the axial contour on the axial rotating accuracy needs further study. Moreover, in the aerostatic spindle system, in addition to the manufacturing error of the spindle rotor, the bearing’s manufacturing error has an impact on the rotating accuracy. Although current engineering practice shows that the bearing’s manufacturing error has less impact on the rotating accuracy than the spindle rotor, the bearing’s manufacturing error may be coupled with that of the rotor, which jointly affects the distribution of the film’s flow field; further research will be needed to evaluate this phenomenon.

## Figures and Tables

**Table 1:** Comparison of average roundness error (RONt) and cylindricity error (CYLt) after simulation processing under different filter parameters.

Item	Filtering Parameters of Measured Data	
	1–5 Gaussian UPR	1–15 Gaussian UPR
Average RONt after machining (μm)	0.047	0.076
CYLt after machining (μm)	0.191	0.278

**Table 2:** Characteristics of the experimental shaft.

Item	Corresponding Parameter
Brand of steel	AISI 1045
Surface heat treatment	Quenching
Surface hardness (HRC)	58–62
Diameter (mm)	100

**Table 3:** Parameters of figuring process.

Item	Corresponding Parameter
Model of the abrasive belt	3M^®^ 261X
Grit size (μm)	5
Grit type	Al_2_O_3_
Frequency of vibration (Hz)	7
Updating speed of the abrasive belt (mm·s^−1^)	10
Contact pressure of the contact wheel (MPa)	0.1

**Table 4:** RONt of corresponding circular section before and after machining.

**Section Number**	**RONt on the Section (** **μm)**	
	**Before Machining**	**After Machining**
Section 1	0.38	0.08
Section 2	0.45	0.11
Section 3	0.39	0.08
Section 4	0.39	0.09
Section 5	0.42	0.10
ection 6	0.46	0.12
Section 7	0.44	0.13
